# The smell of spud-stress: a pilot study testing the viability of volatile organic compounds as markers of drought stress in potato (*Solanum tuberosum*)

**DOI:** 10.3389/fpls.2025.1579611

**Published:** 2025-07-22

**Authors:** Luke Bell, Kala Radha, Dominic Hill

**Affiliations:** School of Agriculture, Policy and Development, University of Reading, Reading, United Kingdom

**Keywords:** gas chromatography mass spectrometry, farnesene, 2-methyldecalin, plant stress, plant biochemistry

## Abstract

**Introduction:**

Volatile organic compounds (VOCs) are products of plant secondary metabolism with the potential for signalling early stress response. This pilot study investigated the potential of VOCs as markers for drought stress in potato. We hypothesised that differences in VOC emissions between cultivars may reflect genotypes with greater adaptive efficiency to drought stress.

**Methods:**

Using thermal desorption collection and gas chromatography-mass spectrometry (GC-MS) techniques, we profiled the VOCs emitted by two potato cultivars, Maris Piper and Désirée, under well-watered and drought conditions, across a four-week period (*n* = 3 per cultivar, treatment, and time-point).

**Results:**

We identified 23 compounds, and tentatively identified another 49 compounds, including sesquiterpenes, alkanes, monoterpenes, and methylbenzenes. Statistical analysis revealed that seven compounds showed significant differences between cultivars and drought/well-watered treatments. Two farnesene isomers, a xylene isomer, 2,6-dimethyldecane, decahydronaphthalene, and 2-methyldecalin were identified as tentative markers of drought stress.

**Discussion:**

Our findings suggest that VOCs could be used for detection of drought stress in potato plants, contributing to improved irrigation management and the breeding of more drought-tolerant varieties. Further research is needed to validate these findings and explore the underlying mechanisms.

## 1 Introduction

Volatile organic compounds (VOCs) are a broad and diverse group of low molecular weight, carbon-based chemicals that plants emit as part of their metabolic processes (Escobar-Bravo et al., 2023). These compounds, which include a wide array of alcohols, aldehydes, ketones, esters, terpenes, and other hydrocarbons, are involved in multiple plant functions, ranging from growth and development (Cofer et al., 2018), to defence mechanisms against pests (Zhou and Jander, 2021) and pathogens (Lal et al., 2018).

VOC emission is initiated by the perception of stress, often leading to the accumulation of signalling molecules like abscisic acid (ABA) and reactive oxygen species (ROS) (Chandrasekaran et al., 2022). ROS can directly contribute to substrate generation for green leaf volatiles (GLVs) via lipid peroxidation and the LOX pathway (Dar et al., 2015). Both ABA and ROS, along with other stress signals, modulate gene expression and enzyme activity within the foundational 2-*C*-methyl-D-erythritol 4-phosphate (MEP) and mevalonic acid (MVA) pathways, increasing the supply of isoprenoid precursors (Meng et al., 2025). These precursors are then acted upon by specific synthases, like terpene synthases (TPS), and other pathway enzymes (e.g., in the phenylpropanoid pathway), whose activities are upregulated by stress signals (Bhatla, 2018). The result is the emission of a complex blend of VOCs tailored to the specific stress encountered.

The role of VOCs in plant physiology has been extensively studied, revealing their significance not only in plant-environment interactions (Ode et al., 2014) but also as indicators of plant stress (Copolovici et al., 2014). Among the various types of stress that plants encounter, drought stress has emerged as a critical focus, particularly in the context of global climate change and the increasing frequency of extreme weather events (Bisbis et al., 2018).

Drought stress is a major abiotic stress factor that severely affects plant growth, productivity, and survival. It is characterised by a lack of sufficient water to support metabolism, which disrupts the plant’s normal physiological processes, leading to reduced photosynthesis, impaired nutrient uptake, and oxidative damage (Giordano et al., 2021). In response to drought, plants activate a series of complex biochemical and physiological mechanisms aimed at conserving water and maintaining cellular function (Gervais et al., 2021). These responses include stomatal closure to reduce water loss, the accumulation of osmoprotectants, the upregulation of antioxidant enzymes, and the modification of root architecture to enhance water uptake (Wahab et al., 2022). Plants produce VOCs constitutively, but are capable of altering their metabolic pathways in response to environmental and biological stimuli, leading to the production and release of certain VOCs in specific circumstances (Salerno et al., 2017). This includes in response to insect herbivory (Moreira and Abdala-Roberts, 2019), fertiliser application (Chen et al., 2008), and combinations of abiotic and biotic factors (Martín-Cacheda et al., 2023).

Potato (*Solanum tuberosum* L.) is one of the most important food crops globally, serving as a staple for millions of people (Agrawal et al., 2024). The crop is highly sensitive to water availability, with drought conditions leading to significant reductions in tuber yield and quality (Obidiegwu et al., 2015). As a shallow-rooted plant, potatoes are particularly vulnerable to fluctuations in soil moisture, making them prone to drought stress (Hill et al., 2021). The impact of drought on potato production is not just a matter of reduced yields; it also affects tuber development (Chang et al., 2018), nutrient content, and the overall health of the plant (Xu et al., 2016). Consequently, there is an urgent need to develop strategies that can help mitigate the effects of drought on potato crops, ensuring food security and the sustainability of agricultural practices. Whist potato has a relatively high water use efficiency, it still requires large volumes of water to grow effectively and attain optimum yields (Monneveux et al., 2013).

Recent advancements in plant physiology and analytical chemistry have highlighted the potential of VOCs as non-invasive biomarkers (Abbas et al., 2023) for detecting drought stress in crops, including potatoes (Frank and Engel, 2013). The emission of VOCs is a dynamic process influenced by environmental conditions (Copolovici et al., 2014), developmental stages (Bell et al., 2020), and stress factors (Agho et al., 2023). Under drought stress, potato plants have been shown to emit a bouquet of VOCs, which can be detected and analysed using gas chromatography mass spectrometry techniques (Cara et al., 2020). Specific drought-induced VOCs could serve as a chemical signature of the plant’s stress response, providing valuable insights into the underlying physiological and biochemical changes, as has been shown in other studies (Copolovici et al., 2014; Weldegergis et al., 2015; Lupitu et al., 2023). For example, terpene and sesquiterpene compounds are emitted from Solanaceae leaf surfaces via glandular trichomes (Gonzales-Vigil et al., 2012). These act as part of the plants’ defence against herbivory and rupture upon disturbance (Bai et al., 2024). In contrast to this mechanism, plants also transmit VOCs via stomata and gas exchange. Under drought conditions, stomata close to reduce water loss, thereby reducing the amount of VOCs emitted via this mechanism (Escobar-Bravo et al., 2023). Previous research has studied the response of potato cultivars to combined biotic and abiotic stress, and there is evidence to suggest that water restriction influences the induced resistance of plants to herbivore attack, and that drought reduces the amounts of VOCs that are emitted (Vázquez-González et al., 2022). A study on tomato plants showed that combined stress may have an additive impact on VOC emissions, and that this can be perceived by unstressed plants that in turn increase their own VOC production (Catola et al., 2018). Many studies analysing VOCs in this context have however been limited by small pot sizes (<5 L) which has been shown to significantly impact the morphology and physiology of plants (Hill et al., 2024a). Well-watered controls of potato plants grown in small pots are unable to maintain adequate soil moisture content, and effectively act more as a slightly reduced drought treatment due to pot-binding effects (Hill et al., 2024a). This makes it difficult to determine what are ‘true’ effects of the interactions being tested, and which are as a result of such confounding experimental effects.

The identification and characterisation of drought-specific VOCs in potato plants grown in suitably sized containers offers several potential applications. Compounds could be used to develop early warning systems for drought stress, allowing for more precise irrigation management and reducing water usage. By monitoring VOC emissions, farmers and agronomists could detect the onset of drought stress before visible symptoms appear, enabling timely interventions to prevent crop loss, and improve the efficiency of irrigation applications. Moreover, understanding the VOC profiles associated with drought stress could contribute to the breeding of more drought-tolerant potato varieties. Selecting for traits that reduce the sensitivity of plants to drought-induced VOC changes could lead to the development of crops better suited to withstand water scarcity and transient drought stress. In addition to their practical applications, studying VOCs in the context of drought stress in potatoes provides fundamental insights into plant stress physiology, such as the specific pathways involved in VOC synthesis, and the regulation of these pathways by plant hormones such as ABA (Yao et al., 2023). Such efforts have been attempted by some studies as a means to remotely and autonomously record plant VOC emissions, using techniques technologies such as e-Nose (electronic-nose) (Murali-Baskaran et al., 2022). This approach shows promise but the numbers of compounds that can be reliably detected and identified using e-Nose is limited compared to mass spectrometry techniques.

This paper explores the potential of VOCs as markers for drought stress in potato plants using thermal desorption collection and gas chromatography mass spectrometry (GC-MS) with chemometric techniques. By profiling the VOCs emitted by potato plants under drought conditions in large containers, this pilot study aimed to identify specific compounds that could serve as reliable indicators of water stress without the known confounding impacts of pot-binding. We hypothesised that sesquiterpene compounds would increase in abundance in response to drought stress through trichome emission, with a corresponding decrease in VOCs carried through stomata as these close in response to water deficit.

## 2 Methods

### 2.1 Plant Materials and growing conditions

**Figure 1:**
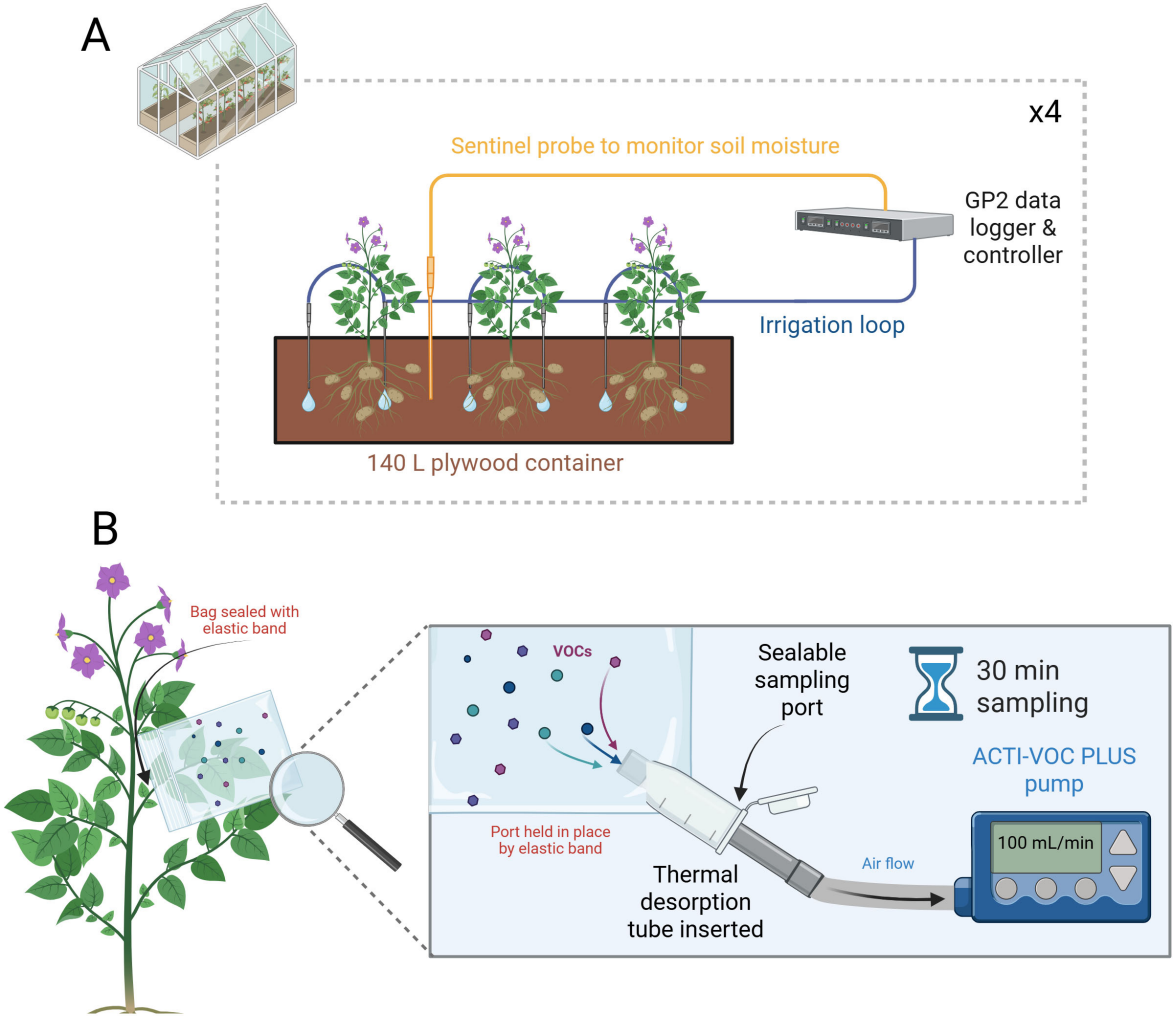
**(A)** Example diagram of the potato plant cultivation setup within the glasshouse environment. Three potato plants were grown per 140 L container with irrigation monitored and controlled by a GP2 control system and WET150 sentinel sensors. Four troughs were set up in this fashion, with two containing Désirée and two containing Maris Piper. Each of the two respective troughs for each cultivar were used as the well-watered and drought treatments, respectively, and supplied by two independently controlled irrigation loops. Upon drought imposition, the irrigation loop to the two drought treatment troughs was halted. **(B)** Potato plant VOCs were collected using a bag enclosing an entire leaf stem. The bag was modified with an Eppendorf tube sampling port, through which thermal desorption (TD) tubes could be inserted without compromising the VOCs inside. Air was drawn through the TD tube by a battery-powered ACTI-VOC PLUS pump for 30 mins, at a flow rate of 100 mL/min. Created in https://BioRender.com.

Potato plants were grown in a pot experiment at the Crop & Environment Laboratory (51°26’13.0”N 0°56’31.0”W) at the University of Reading, UK, according to the methods and conditions presented by Hill et al (Hill et al., 2024b). Pots consisted of twelve bespoke plywood troughs (1,140 x 300 x 412 mm; L x W x H). Each trough was filled with 148 L of a 2:1 volume mixture of John Innes No. 2 compost and sharp sand (Jubilee Building Supplies, Bracknell, UK; **Figure 1A**). Each trough was premixed and fertilised with 576 g of Osmocote Pro 3-4M (Everris, Geldermalsen, Netherlands).

18 pre-sprouted seed tubers of cultivars Maris Piper and Désirée were planted at a depth of 10 cm, with three tubers per trough. Tubers were pre-treated with Imazalil fungicide and were provided by Branston Ltd. (Lincoln, UK). Plants were initially grown outdoors and uncovered until 65 days after planting (DAP), before being moved into a glasshouse compartment. While outdoors, plants were rainfed, which was sufficient to maintain a well-watered condition.

In the glasshouse environment, plants were grown under LED lights with a 16-hour photoperiod, and irrigation was controlled by a GP2 data logger and four WET150 sensors (Delta-T Devices, Cambridge, UK). Each WET150 sensor was buried at a depth of 30 cm at a 60° angle relative to the soil surface. One sensor for each cultivar and treatment was used as a sentinel controlling irrigation for each of the other corresponding troughs. Well-watered and water-restricted troughs were each connected to one of two irrigation loops. Each loop was independently controlled by the GP2 based on the soil moisture content (**Figure 1A**). Both loops supplied each trough with 12 L of water per hour via two drippers per plant (six per trough). Before the onset of water restriction, the GP2 was programmed to check each probe every hour for soil moisture content (SMC). An irrigation threshold of <36% SMC was used based on the following calibration values: A_0_ = 1.32, A_1_ = 8.70. If the condition was met by both probes within a treatment, irrigation was turned on automatically. The GP2 then re-checked each sensor for SMC every minute until ≥36% was reached and then irrigation was stopped. A SMC of 36% was chosen based on WET150 readings at 80% soil water capacity, which was calculated gravimetrically (Turner, 2019). Well-watered conditions were maintained until 69 DAP. On this date the irrigation loop for water-restricted troughs was manually turned off, with water-restricted conditions maintained for the remainder of the experiment. The treatment of well-watered plants remained the same throughout. Treatments ceased at 96 DAP. Physiological data pertinent to the plants tested in this study can be found in Hill et al. (2024b).

### 2.2 Volatile organic chemical collection

VOCs were collected from leaves and stems of potato plants in triplicate for each cultivar and treatment (*n* = 3) over a period of four weeks after the imposition of drought in the experiment (69 DAP). The same plant from each cultivar/treatment trough was sampled each week. Mature lateral stems at approximately the mid-way point of the total height of the plants were selected for sampling, and whenever possible, the same stem was sampled each week. The sampling protocol was adapted from Bell et al (Bell et al., 2016): one large impermeable oven bag was placed over the mature stem and leaves, and sealed at the end using an elastic band. The bag and stem were supported with a cane and clamp and left to equilibrate for ten minutes before sampling. Active sampling was conducted using preconditioned stainless steel thermal desorption (TD) tubes with a dual Tenax/Sulficarb sorbent (Markes International Ltd., Bridgend, UK). Tubes were conditioned at 240°C for 20 minutes prior to sampling using the Turbomatrix ATD system (Perkin Elmer, Beaconsfield, UK) described in the next section. A sampling port was made in the oven bags by cutting off one corner and inserting an Eppendorf tube (with the end removed). TD tubes were inserted into the port opening and connected to a pre-calibrated ACTI-VOC PLUS pump (Makes International) set to 100 mL per min. Lateral stems were sampled for 30 mins and TD tubes were sealed using brass storage caps until analysis (**Figure 1B**). The sampling duration was based on previously reported methods (Kask et al., 2016; Kivimäenpää et al., 2016), and 30 minutes was chosen as the optimum after test extractions lasting 30, 45, and 60 minutes. To account for possible background environmental VOCs, blank TD tube samples were taken each week by sampling empty oven bags in the glasshouse environment for the same duration. All tubes were sealed using brass long-term storage caps and were analysed the day after sampling.

### 2.3 Gas chromatography mass spectrometry

Gas Chromatography Mass Spectrometry (GC-MS) was performed with an Agilent 7890A-5975C (Stockport, UK) instrument, coupled with a Turbomatrix ATD system for TD tube sample introduction. TD tubes were desorbed at 300°C with a heating rate of 40°C/sec. Separation was achieved using a DB5 column (30 m x 250 μm i.d. x 1 μm; Agilent). The time-temperature program was a ramp of 4°C/min to 300°C, with a final hold of 5 minutes. Carrier gas was helium at 1 mL/min. The MS was operated in electron ionisation (EI) mode at a voltage of -70 eV, with a source temperature of 230°C. A scan range of *m/z* 29–450 was used, with a scan time of 0.7 sec. Data were acquired using Agilent ChemStation. Linear Retention Index (LRI) values were obtained by running an *n*-alkane standard mix (C_5_-C_25_; Merck Life Science UK Limited, Gillingham, UK) in diethyl ether via liquid injection. Compounds were identified by comparing their mass spectra with NIST Mass Spectral Database (v.2020), a custom authentic compound library, and literature LRI values. A threshold of >80 quality score was used for assessing mass spectra identifications. Compounds where no matching RI could be found were classed as tentative identifications (**Table 1**). Data for each compound are presented as normalised peak area values.

**Table 1:** Volatile organic compounds identified in the headspace of two potato cultivars (Maris Piper and Désirée) under well-watered and drought conditions.

Rt	Compound identification	Identification method ^$^	Compound class	Spectra quality	CID number	CAS No.	LRI	Database/literature LRI	References	Friedman test (cultivar + treatment) significance	Friedman test (week + treatment) significance
1.77	<unknown 1>						416			ns	*
2.00	<unknown 2>						482			ns	ns
2.07	<unknown 3>						500			ns	ns
2.09	<unknown 4>						605			ns	ns
2.11	<unknown 5>						608			ns	ns
2.23	<unknown 6>						613			ns	ns
2.55	<unknown 7>						627			ns	ns
2.39	<unknown 8>						636			ns	ns
2.50	<unknown 9>						650			ns	ns
2.60	<unknown 10>						678			ns	ns
3.06	<unknown 11>						714			ns	ns
5.91	P-Xylene	B	Methylbenzene	93	7809	106-42-3	867	865		ns	ns
6.44	Xylene (isomer 2)	B	Methylbenzene	89	7237	95-47-6	894	888		ns	ns
7.28	(+)-α-Pinene	B	Terpene	89	82227		921	917	(Agho et al., 2023)	ns	ns
7.30	Tricyclene	B	Monoterpene	95	79035		922	926		ns	ns
7.44	<unknown 13>						925			ns	ns
7.74	α-Pinene	B	Terpene	93	6654	80-56-8	935	935	(Agho et al., 2023)	ns	ns
8.39	<unknown 14>						956			ns	ns
9.01	1-Ethyl-2-methylbenzene	B	Methylbenzene	84	11903	611-14-3	973	971		ns	ns
9.02	β-Terpinene	C	Monoterpene	91	66841	99-84-3	974			ns	ns
9.83	β-Pinene	A	Terpene	95	14896	127-91-3	992	990	(Agho et al., 2023)	ns	ns
9.79	(-)-β-Pinene	B	Terpene	88	24848167	18172-67-3	997			ns	ns
10.45	α-Terpinene	A	Monoterpene	95	7462		1016	1016	(Agho et al., 2023)	ns	ns
10.60	<unknown 19>						1022			ns	***
10.64	p-Cymene	B	Monoterpene	88	7463	99-87-6	1023	1025		*	**
10.76	M-Cymene	A	Monoterpene	93	10812	535-77-3	1025	1026		ns	ns
10.90	D-Limonene	B	Terpene	99	440917	5989-27-5	1029	1030	(Ali et al., 2022)	ns	*
10.96	2-Ethylhexan-1-ol	A	Alcohol	80	7720		1030	1030		*	ns
11.66	Decahydronaphthalene (isomer 1)	C	Bicyclic hydrocarbon	97	7044		1052			ns	***
11.86	5-Methyldecane	C	Alkane	90	93071		1057			ns	**
11.95	γ-Terpinene	A	Monoterpene	92	7461		1058	1058	(Zhao et al., 2022)	ns	ns
11.96	4-Methyldecane	C	Alkane	91	17835		1060			ns	***
12.09	2-Methyldecane	C	Alkane	94	23415		1064			ns	***
12.32	3-Methyldecane (isomer 1)	C	Alkane	93	92239		1070			ns	***
12.79	<unknown 21>						1083			ns	ns
13.00	Terpinolene	A	Monoterpene	95	11463		1087	1087		ns	ns
13.40	Undecane	A	Alkane	97	14257		1100	1100	(Kate et al., 2023)	ns	**
13.68	2-Methyldecalin	C	Bicyclic hydrocarbon	97	94249		1108			ns	**
14.03	2,6-Dimethyldecane	C	Alkane	93	139395		1118			ns	ns
14.30	1-Methyldecahydronaphthalene	C	Bicyclic hydrocarbon	96	34193		1123			ns	***
14.36	3,7-Dimethyldecane	C	Alkane	81	28468		1127			ns	ns
14.66	1,6-Dicyclohexylhexane	C	Alkane	81	123123		1133			ns	*
15.14	cis,cis-Bicyclo[4.4.0]decane, 3-methyl	C	Bicyclic hydrocarbon	96	6427441		1146			ns	ns
15.38	<unknown 22>						1155			ns	ns
15.49	5-Methylundecane	C	Alkane	90	94213		1156			ns	*
15.72	3-Methyldecane (isomer 2)	C	Alkane	97	92239		1164			ns	*
15.95	3-Methylundecane	C	Alkane	81	13845		1170			ns	*
17.36	Dodecane	A	Alkane	97	8182	112-40-3	1200	1200		ns	*
17.59	Decanal	A	Aldehyde	91	8175	112-31-2	1207	1207	(Duckham et al., 2002)	ns	ns
17.55	2,6-Dimethylundecane	C	Alkane	91	28453		1215			ns	*
20.92	Tridecane	A	Alkane	97	12388	629-50-5	1300	1300	(Mandin et al., 1999)	ns	ns
21.96	<unknown 23>						1339			ns	ns
22.39	α-Cubebene	B	Sesquiterpene	96	442359		1351	1351	(Szafranek et al., 2005)	ns	ns
23.28	Copaene	A	Sesquiterpene	99	19725	3856-25-5	1378	1379	(Morris et al., 2011)	ns	ns
23.59	(-)-cis-β-Elemene	C	Sesquiterpene	96	6431151		1387			ns	ns
23.69	<unknown 24>						1392			*	ns
23.83	β-Elemene	A	Sesquiterpene	95	6918391	33880-83-0	1394	1394	(Khalilov et al., 1999)	ns	ns
23.94	Longipinene (isomer 1)	C	Sesquiterpene	96	520957		1397			ns	ns
24.06	Tetradecane	A	Alkane	97	12389	629-59-4	1400	1400		ns	ns
24.18	<unknown 25>						1404			ns	ns
24.55	<unknown 26>						1408			ns	***
24.41	(-)-α-Gurjunene	B	Sesquiterpene	99	521243	489-40-7	1411	1413		ns	ns
25.02	Caryophyllene (isomer 1)	A	Sesquiterpene	99	5281515	87-44-5	1422	1421	(Weissbecker et al., 2000)	ns	ns
25.13	Caryophyllene (isomer 2)	A	Sesquiterpene	99	5281515	87-44-5	1426	1424		ns	ns
25.52	α-Bergamotene, (E)-(-)-	A	Sesquiterpene	97	6429302	13474-59-4	1438	1438	(Szafranek et al., 2005)	ns	ns
25.48	<unknown 27>						1444			ns	ns
25.41	Farnesene (isomer 1)	B	Sesquiterpene	95	5281517		1445	1446	(Szafranek et al., 2005)	*	ns
25.82	Humulene	A	Terpene	98	5281520	6753-98-6	1455	1457	(Weissbecker et al., 2000)	ns	ns
25.96	Farnesene (isomer 2)	B	Sesquiterpene	97	5281517	18794-84-8	1458	1459	(Szafranek et al., 2005)	ns	ns
26.06	(+)-Aromadendrene	A	Sesquiterpene	99	11095734	489-39-4	1463	1460	(Duckham et al., 2001)	ns	ns
26.08	Longipinene (isomer 2)	C	Sesquiterpene	86	520957		1466			ns	ns
26.10	β-Acoradiene	A	Sesquiterpene	94	20055537		1466	1466		**	ns
26.22	<unknown 28>						1468			ns	ns
26.78	trans-Cadina-1(6),4-diene	A	Bicyclic hydrocarbon	91	10798255	20085-11-4	1477	1475		ns	ns
26.57	Muurolene (isomer 1)	B	Sesquiterpene	97	12313020	30021-74-0	1478	1479	(Karlsson et al., 2009)	ns	***
27.02	Muurolene (isomer 2)	B	Sesquiterpene	96	12313020	30021-74-0	1485	1486		ns	***
27.12	<unknown 29>						1488			ns	*
27.15	α-Santalol	C	Sesquiterpene	95	60970		1496			ns	ns
27.22	(+)-Cadinene (isomer 1)	B	Sesquiterpene	95	6432404	39029-41-9	1499	1497	(Karlsson et al., 2009)	ns	ns
27.26	Muurolene (isomer 3)	B	Sesquiterpene	99	12306047		1502	1499		ns	ns
27.50	Cuparene	B	Sesquiterpene	98	86895	16982-00-6	1508	1505		ns	ns
27.86	β-Bisabolene	A	Sesquiterpene	96	10104370	495-61-4	1511	1511	(Khalilov et al., 1999)	ns	ns
27.97	(-)-β-Curcumene	B	Sesquiterpene	86	14014430	28976-67-2	1512	1509		ns	ns
28.04	Muurolene (isomer 4)	B	Sesquiterpene	97	101708	483-75-0	1517	1519		ns	**
27.97	cis-β-Guaiene	C	Sesquiterpene	93	15560253		1523			ns	ns
28.05	Cadina-1(10),4-diene	B	Sesquiterpene	95	10223		1525	1524		ns	***
27.97	(+)-δ-Cadinene	A	Sesquiterpene	97	441005		1526	1524	(Szafranek et al., 2005)	ns	**
28.07	(+)-Cadinene (isomer 2)	B	Sesquiterpene	92	6432404		1529	1530	(Karlsson et al., 2009)	ns	*
28.26	β-Curcumene	C	Sesquiterpene	89	6428461		1535			**	ns
28.49	<unknown 30>						1540			ns	ns
28.77	<unknown 31>						1541			ns	ns
28.80	Aromadendrene	C	Sesquiterpene	98	91354		1553		(Khalilov et al., 1999)	ns	ns
29.22	<unknown 32>						1555			ns	ns
28.98	<unknown 33>						1559			ns	ns
29.44	(1aR,4R,7R,7aS,7bR)-1a,2,3,4,6,7,7a,7b-Octahydro-1,1,4,7-tetramethyl-1H-cycloprop[e]azulene	C	Sesquiterpene	96	11009053		1571			ns	ns
29.98	Muurolene (isomer 5)	B	Sesquiterpene	96	12313020	30021-74-0	1580	1586	(Karlsson et al., 2009)	ns	ns
29.76	5,9-Undecadien-1-yne, 6,10-dimethyl-	C	Alkyne	81	549649		1581			ns	ns
29.92	10s,11s-himachala-3(12),4-diene	C	Sesquiterpene	95	14038471		1586			ns	ns
30.34	Hexadecane	A	Alkane	94	11006	544-76-3	1600	1600	(Kate et al., 2023)	ns	ns
30.82	<unknown 34>						1608			ns	ns
31.16	<unknown 35>						1620			ns	ns
31.14	1,4-Methanobenzocyclodecene, 1,2,3,4,4a,5,8,9,12,12a-decahydro-	C	Methylbenzene	92	556414		1627			ns	*
31.91	<unknown 36>						1646			ns	ns
32.32	<unknown 37>						1660			ns	ns
33.09	Isoledene	C	Sesquiterpene	97	530426	95910-36-4	1694			ns	ns

N.B. Compounds are ordered according to Linear Retention Index (LRI) values. Significance levels from Freidman’s test are presented for each compounds according to cultivar x treatment and sampling week x treatment.

$ - Identification methods: A = Identification, LRI and spectra match with authentic standard; B = Tentative identification, LRI and spectra match with literature source and/or NIST20 library; C = Tentative identification, NIST20 library match, no literature LRI found.

Significance values according to Freidman tests: not significant = ns; p<0.05 = *; p<0.01 = **; p<0.001 = ***.

### 2.4 Chemometrics and statistical analysis

Peak area data were exported from ChemStation, collated and normalised. Compound data underwent four tests for normality, comprising Shapiro-Wilk, Anderson-Darling, Lilliefors, and Jarque-Bera tests. Each consistently showed the data had a non-normal distribution. Friedman’s test (one-tailed) was selected for analysis as it is (i) a non-parametric test, (ii) accounts for repeated measures, and (iii) is appropriate for independent samples. An accompanying Nemenyi’s procedure (two-tailed) was conducted in order to determine any significant multiple pairwise comparisons between treatments/cultivars. Statistical significance was defined at the *p* = ≤0.05 level. These analyses were conducted using XLstat (Addinsoft, Paris, France).

Chemometrics and Principal Component Analysis (PCA) were performed using ChromCompare+ (CC+) within ChromSpace (v. 2.1.7.; Markes International Ltd.). Raw MS data files were converted to.lsc format for analysis and aligned using the automatic alignment algorithm. Aligned data files were processed using the Dynamic Background Correction (DBC) algorithm using a peak width of 5 sec. Processed files then underwent integration using a tile sum approach, with a retention time window of 5 sec and 25% overlap. Raw ion data were processed and filtered to identify ions explaining the largest amount of discrimination (Spadafora et al., 2023) between cultivars and treatments. Ion features were initially filtered using a minimum intensity cut-off of 2,500 counts. The remaining ion features were normalised using Probabilistic Quotient Normalisation (PQN) based on the mean ion abundance across all samples (Gorrochategui et al., 2016). The normalised data were then filtered using the feature discovery algorithm for the top 50 discriminating ions between experimental treatments, cultivars, and time points. These features’ retention times were then cross-referenced with DBC chromatograms in ChromSpace to identify the associated chromatogram peaks. The *m/z* of the ions identified by CC+ were required to be present in both the observed mass spectra and the library mass spectra for a reliable association to be determined.

## 3 Results

### 3.1 Compound identification

Across the two cultivars and four sampling weeks 103 peaks were observed (**Table 1**). Of these, 31 could not be reliably or tentatively identified and were designated as ‘unknown’. The 72 compounds that were identified, or tentatively identified, were comprised of 33 sesquiterpenes, 16 alkanes, seven monoterpenes, six terpenes, five bicyclic hydrocarbons, three methylbenzenes, one alcohol, one aromatic aldehyde, one ester, one aldehyde, and an alkyne (**Table 1**). 23 compounds could be reliably identified by matching spectra and LRI values with authentic compounds. Tentative identifications were assigned to the remaining 49 compounds. Peaks found in only one sample of the three sample replicates collected were not included in the analysis.

### 3.2 Effects of water restriction on potato cultivar VOC abundance

**Figure 2:**
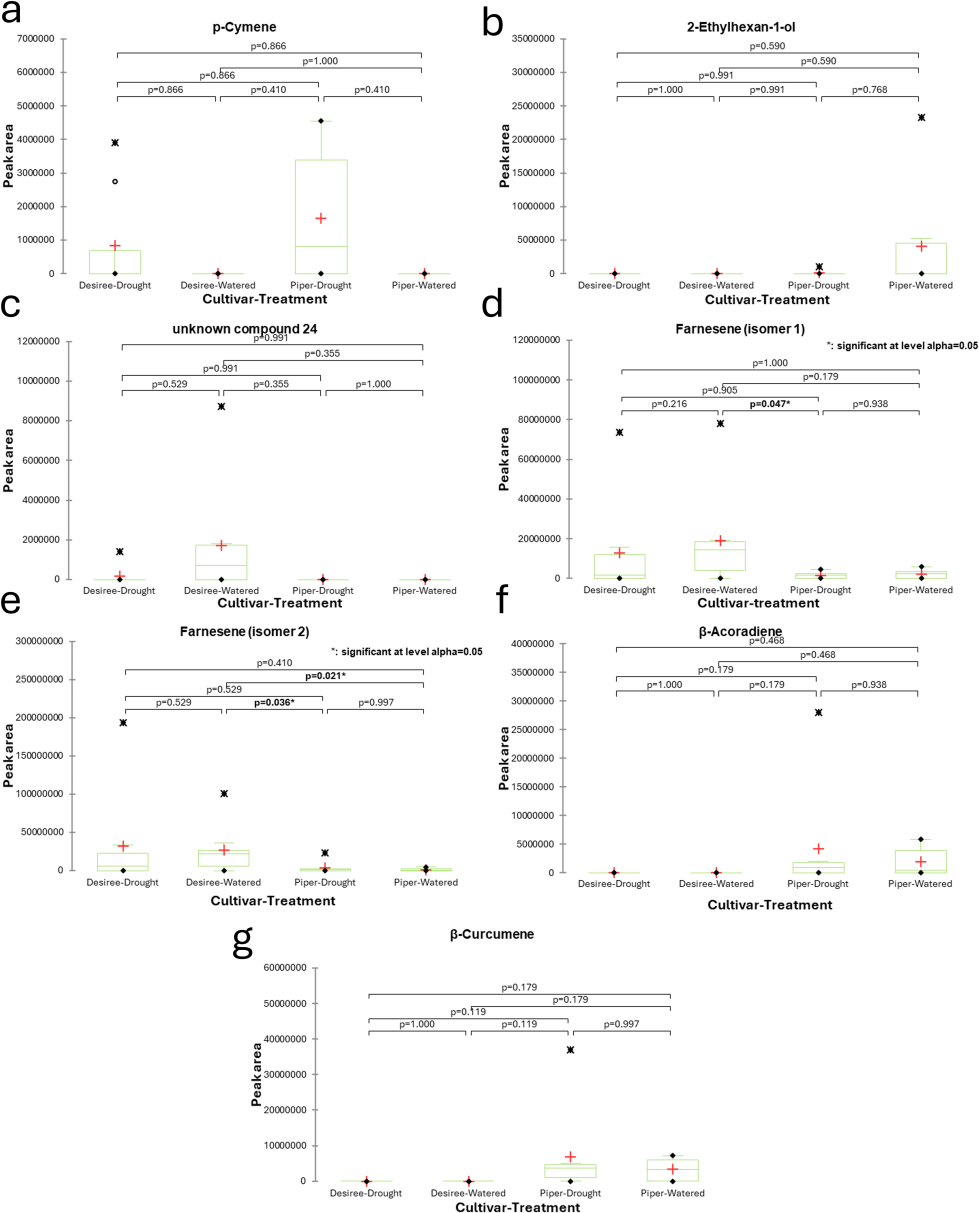
Boxplots displaying the average normalised peak areas for VOC compounds identified from potato plant headspace extracts under well-watered and drought treatments over a four-week period. The cultivars tested were Désirée and Maris Piper. p-cymene **(a)**, 2-ethylhexan-1-ol **(b)**, unknown compound 24 **(c)**, farnesene isomer 1 **(d)**, farnesene isomer 2 **(e)**, β-acoradiene **(f)**, and β-curcumene **(g)** displayed significant differences between samples when tested using Friedman’s test (*p* = ≤0.05) but only farnesene isomers were significantly different at the pairwise comparison level (Nemenyi’s procedure; *p* = ≤0.05). Significant differences are indicated in bold text. Green boxes represent the sample distribution about the mean, minus outliers (upper quartile, median, lower quartile); error bars represent minimum and maximum values, minus outliers; red + represent the sample mean, including outliers; black • represent minimum and maximum sample peak area values; black X represent outliers.

Statistical analysis of the VOC data using Friedman’s test revealed significant differences in the levels of seven compounds when comparing different plant cultivars and watering conditions (drought vs. well-watered): p-cymene, 2-ethylhexan-1-ol, an unidentified compound (24), farnesene (isomer 1), farnesene (isomer 2), β-acoradiene, and β-curcumene. However, a closer examination using Nemenyi’s procedure for pairwise comparisons showed that only the two farnesene isomers had statistically significant differences. Specifically, farnesene isomer 1 levels were significantly different between the Maris Piper cultivar under drought conditions and the well-watered Désirée cultivar. Farnesene isomer 2 levels showed significant differences when comparing the well-watered Désirée cultivar to both the well-watered and the drought-stressed Maris Piper treatments. These results are presented in **Figure 2**. Samples were also analysed for changes over time between the four, weekly sampling points to determine if responses to drought manifested at specific times. Several compounds showed significant changes in abundance (**Table 1**), however none of the pairwise comparisons yielded significant differences between well-watered and droughted plants within the same sampling week. Generally, VOCs were seen to peak in week 3 of sampling and began to decline in week 4.

### 3.3 Chemometrics analysis

**Figure 3:**
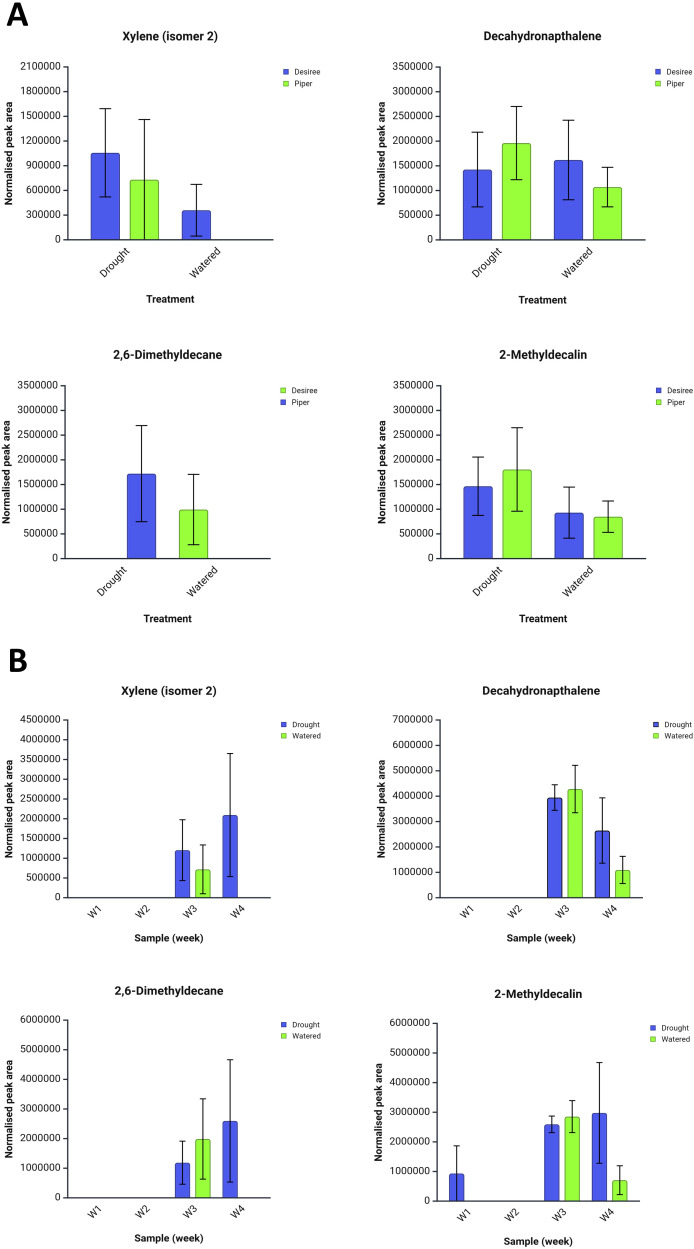
Total average normalised peak areas for VOC compounds identified by ChromCompare+ chemometrics software from potato plant headspace extracts under well-watered and drought treatments over a four-week period. **(A)** Effects of drought and well-watered treatments on two cultivars, Désirée and Maris Piper. **(B)** Effects of drought and well-watered treatments over a four week sampling period. Ions associated with these compounds were found to be discriminatory between experimental treatments. Error bars represent the standard error of the mean. Created in https://BioRender.com.

Of the top 50 ion features six could be reliably associated with compound mass spectra. These were xylene isomer 2 (*m/z* 105), decahydronaphthalene (*m/z* 69), 2,6-dimethyldecane (*m/z* 71), and 2-methyldecalin (*m/z* 123). Corresponding total peak area data for these compounds are presented in **Figure 3** according to cultivar and drought treatment, averaged across the four sampling weeks. None of these compounds were significantly different between treatments according to Friedman’s test and CC+ chemometrics, however some trends are apparent within the data. Xylene isomer 2 was discriminatory between well-watered and drought conditions, only being produced by plants under the drought treatment (both Maris Piper and Désirée). A distinct trend was also observed for 2-methyldecalin with peak areas being higher under the drought treatment for both cultivars. Assuming that these compounds are of natural origin within the plants, they may serve as potential drought precursor markers. Through utilisation of targeted GC-MS approaches this could constitute a viable non-destructive drought detection pathway.

**Figure 4:**
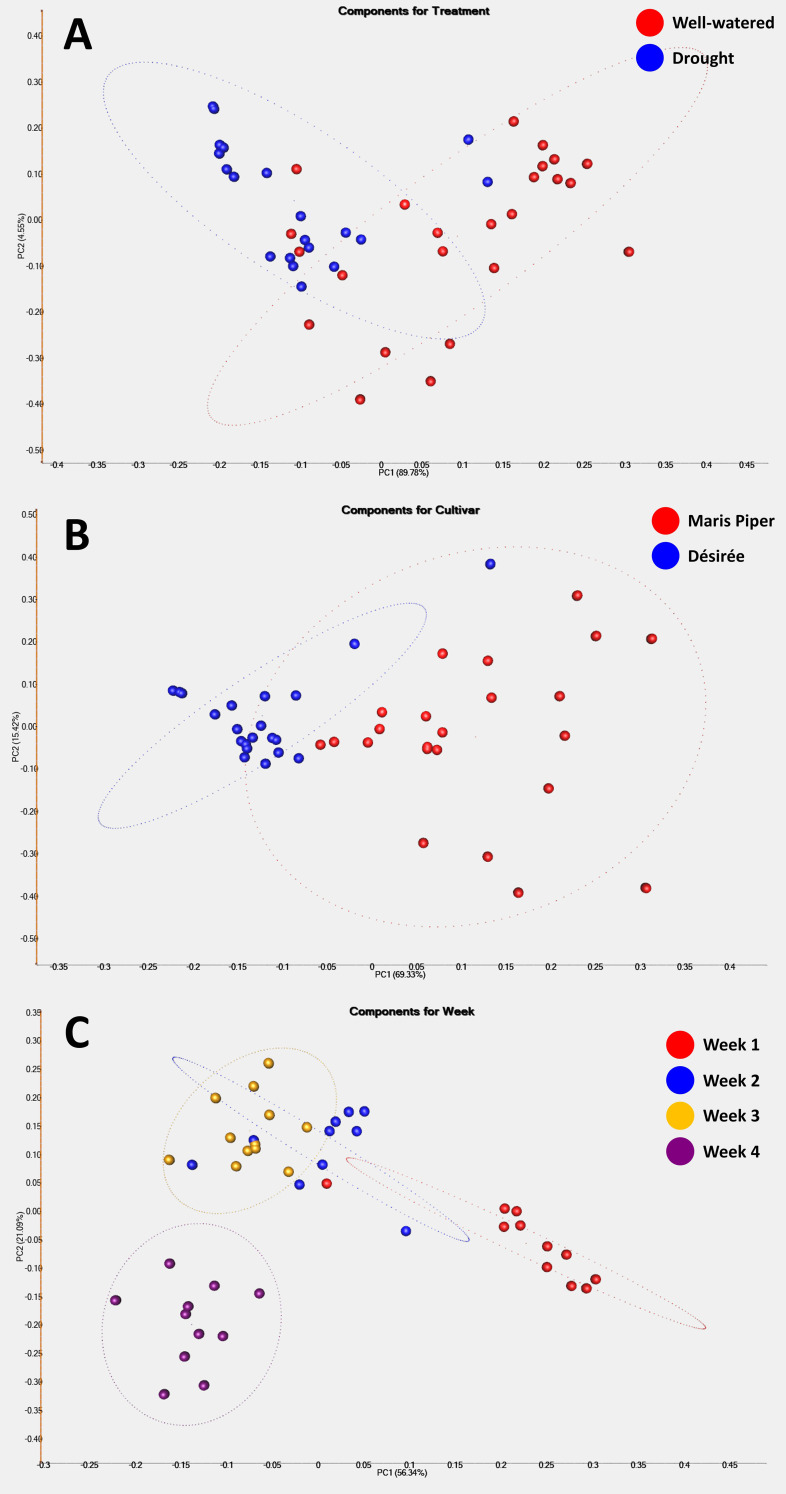
Principal Component Analysis of the top 50 discriminating ions features observed between experimental treatments (**(A)** well-watered vs. drought), cultivars (**(B)** Désirée vs. Maris Piper), and time points (**(C)** weeks 1-4). Principal Components 1 and 2 were selected for presentation in each analysis as these explained the largest proportions of total variance between the samples. PC1 and PC2 explained 89.9% and 4.6% of the data variance, respectively, for experimental treatment **(A)**. PC1 and PC2 explained 69.3% and 15.4% of the data variance, respectively, for cultivar **(B)**. PC1 and PC2 explained 56.3% and 21.1% of the data variance, respectively, for sampling week **(C)**.

PCA analysis was used to determine underlying spatial relationships within the data, based on the top 50 discriminating ions observed between treatments (well-watered vs. drought), cultivars (Désirée vs. Maris Piper), and time points (weeks 1-4) (**Figure 4**). According to treatment, only a weak separation of samples could be identified (**Figure 4A**), with droughted samples tending to form a tighter cluster than well-watered samples. This perhaps indicates that VOC response under drought is less variable compared with when plants are un-stressed, and as a means of conserving metabolites. Results according to cultivar produced a stronger, but not complete separation (**Figure 4B**), with Désirée samples being more uniform than Maris Piper. This is reflected somewhat in **Figure 2**, where Maris Piper generally had a lower overall abundance of VOCs compared to Désirée. The strongest separations observed were according to the sampling week (**Figure 4C**) where there is a clear progression of clusters from week 1 (red) through to week 4 (purple). As would be expected, this reflects the progression and evolution of the potato VOC profile over time as plants grow, however this natural ‘noise’ may make development of a universal potato drought marker challenging.

## 4 Discussion

### 4.1 Differential VOC emissions in potato cultivars under drought

The differential emission of VOCs by potato cultivars under drought stress is a multifaceted phenomenon governed by an intricate interplay of physiological adaptations and genetic determinants (Vázquez-González et al., 2022). Drought alters potato physiology, impacting stomatal conductance, photosynthetic capacity, oxidative balance, osmotic regulation, and root system architecture (Wahab et al., 2022). These physiological changes influence the availability of precursors for VOC biosynthesis, the activity of relevant enzymatic pathways (isoprenoid, lipoxygenase, and phenylpropanoid), and the physical mechanisms of VOC release (Murali-Baskaran et al., 2022).

In this paper 103 compound peaks were detected, with 23 positive identifications and 49 tentative identifications made. The methodological approach is simple and non-destructive, meaning that the same plants (and leaves and stems on those plants) can be sampled on multiple occasions, so that changes in response to drought stress can be recorded. The experimental data collected over a four-week period has revealed compounds that could be used as markers of drought stress in potato plants with more extensive investigation. Importantly, this study was conducted on plants that were not subject to the restrictions of small pot sizes and pot binding that have confounded previous studies.

As this was a pilot experiment, the statistical power was limited, and so further work is required to explore these observations more deeply. This would necessitate larger sample sizes across multiple locations and environments to determine if observations are stable. Cultivation in a glasshouse environment with supplementary LED lighting may impact VOC emissions compared to field grown crops with only natural light, for example (Arena et al., 2016). It would also require testing of additional potato cultivars. As has been shown here, there may be differential responses to drought according to genotype. Maris Piper is classed as a drought susceptible cultivar, and Désirée as (relatively) drought tolerant (Hill et al., 2024b). In a previous paper conducted on the same plants as this study (Hill et al., 2024b) it was observed that drought treatment had significant effects on fresh tuber yields. There were no significant differences observed in yields between the two cultivars for each treatment, but both were significantly impacted by the imposition of water-restriction. According to the evidence from these experiments, it is apparent that there is little drought tolerance displayed by either Maris Piper or Désirée under these experimental conditions.

### 4.2 The genetic and mechanistic basis of cultivar-specific VOC responses to drought in potato

Genetic variation between potato cultivars is the primary driver of differential VOC responses. While there were few statistically significant differences observed between Maris Piper and Désirée in this study, the resulting variation may manifest as a result of different factors. For example, cultivars differ in their inherent ability to manage water deficit through more efficient stomatal control, resilient photosynthetic machinery, robust antioxidant systems, effective osmotic adjustment, or superior root system architecture for water acquisition (Haghpanah et al., 2024). These physiological differences lead to varying degrees of stress experienced at the cellular level, which in turn dictates the nature and intensity of VOC emissions (Widhalm et al., 2015). Allelic diversity in structural genes encoding enzymes of the isoprenoid (e.g., terpene synthases like *StKS*) (Kutty and Mishra, 2023), lipoxygenase (*StLOX* family) (Zhu et al., 2024), and phenylpropanoid (e.g., *PAL*, *CHS*) pathways (Payyavula et al., 2012) directly affects the type and quantity of VOCs produced. Cultivar-specific expression patterns of these genes under drought further contribute to unique VOC signatures (Murali-Baskaran et al., 2022), however it is unclear how xylene and 2-methyldecalin might fit into this picture, as they are not known to be products of the main VOC biosynthesis pathways.

Genetic differences in regulatory elements, such as transcription factors (e.g., *MYB*, *WRKY*) and components of signalling pathways (notably ABA signalling, involving genes like *HAB1*) (He et al., 2021), may also play a crucial role in cultivar-specific drought responses. These regulators modulate the expression of VOC biosynthetic genes and coordinate broader stress responses, leading to cultivar-specific VOC profiles. Epigenetic modifications also appear to contribute an additional layer of regulatory diversity (Abbas et al., 2023).

The interplay between these physiological and genetic factors means that VOC profiles are not merely passive by-products of stress but represent active adaptive responses (Shafi et al., 2024). Some VOCs may offer direct protection (e.g., as antioxidants) (Catola et al., 2018), while others serve as signals (Moreira and Abdala-Roberts, 2019). The balance between stress-induced damage volatiles and actively synthesised protective/signalling volatiles likely differs between tolerant and sensitive cultivars.

Potato shares fundamental VOC response mechanisms with other plants, including the involvement of common VOC classes (terpenoids, GLVs, and phenylpropanoids/benzenoids), regulation by key phytohormones (e.g., ABA, jasmonic acid, salicylic acid, ethylene) (Kutty and Mishra, 2023). These play a critical role in stomatal conductance and gating VOC emissions, with oxidative stress as a primary trigger (Lupitu et al., 2023). Conserved gene families like *LOX*, *CYP450s*, and *MYB* transcription factors are implicated in potato drought response, as they are in many other species (Zeng et al., 2025). However, potato also shows distinctive features. The balance between different hormonal signalling pathways (e.g., ABA/ethylene versus JA) appears to be highly cultivar-dependent and linked to drought tolerance strategies like rooting depth (Obidiegwu et al., 2015). A key finding is the significant attenuation of herbivore-induced VOCs (HIPVs) under drought, primarily due to stomatal closure, which has considerable ecological implications for plant-insect interactions (Shafi et al., 2024). Potato’s shallow root system likely leads to rapid stress perception and an early VOC response (Martín-Cacheda et al., 2023), while the strong metabolic sink of developing tubers may impose constraints on the resources available for sustained VOC synthesis in the foliage (Nogia and Pati, 2021). This potentially leads to an acute reaction and conservation strategy for VOC emissions.

Comparatively, other Solanaceae like tomato have more detailed VOC profiles documented under combined stresses, and often showing synergistic increases in specific terpenes and benzenoids (Baba et al., 2025). Cereal crops such as wheat exhibit distinct VOC profiles under drought (Camaille et al., 2021), and specific compounds like benzoxazinoids are produced in maize (Sutour et al., 2024). Model plants like *Arabidopsis* have provided crucial insights into the function of specific genes and the impact of microbial VOCs on drought tolerance (Liu and Zhang, 2015). By contrast, woody plants often possess VOC storage structures and deeper root systems, and can display different emission kinetics (Ninkovic et al., 2021). As a consequence they have a greater decoupling of emission from immediate photosynthetic activity compared to herbaceous plants like potato, which rely more on *de novo* synthesis (He et al., 2025).

### 4.3 Key potato volatiles identified in the pilot study

Farnesene isomers are well known VOCs produced by potato plants (Szafranek et al., 2005) and act as attractants to pest predators (such as the stinkbug *Perillus bioculatus*) when released (Weissbecker et al., 2000). β-farnesene has also been previously highlighted by Vázquez-González et al. (2022) as a prominent VOC in potato response to combined water and herbivore stress (despite the possible confounding effects of small 4 L pot size).

Xylene isomers are known to act as attractants to pest natural enemies (Duc et al., 2022) and may be potential markers for pathogen infestation (Steglińska et al., 2022). It should be noted that xylene isomers are common synthetic by-products found in tars, but they have been routinely reported as having biological origins in potato plants (Steglińska et al., 2022), potato-based products (Duckham et al., 2001; Majcher and Jeleń, 2005; Zhao et al., 2022), and related Solanaceae species (Suarez and Duque, 1991; Luning et al., 1994). The origins of xylene within plants is not well described.

Similarly, very little is known about the origin and function of 2-methyldecalin, and to our knowledge, its presence has not been previously reported in potato. It has been reported as part of the VOC fraction of neem (*Azadirachta indica*) and has structural similarities with other well-known biological bicyclic sesquiterpene compounds, such as geosmin, for example. It likewise cannot be discounted that the compound has a synthetic origin, however we took all necessary steps in order to mitigate the reporting of any environmental or synthetic compounds. This consisted of taking ‘blank’ samples from empty collection bags inside the glasshouse environment where the potato plants were growing. The compounds identified within these environmental control samples were removed from the analyses and are not included in **Table 1**.

We have tentatively identified two farnesene isomers, xylene, decahydronaphthalene, 2,6-dimethyldecane, and 2-methyldecalin as potential markers for drought stress of potato in this study. This was based on the best data and information available, but further work will be required to confirm our observations. With further research and development it may be possible to utilise these VOCs as phenotypic chemical markers for assisted selection of drought tolerant potatoes.

### 4.4 Conclusions

While the complexity and cultivar-specificity of VOC responses pose challenges for identifying universal biomarkers for drought tolerance in potato, they also offer opportunities. Understanding the genetic basis of desirable VOC profiles – those associated with enhanced physiological resilience or effective stress signalling – can inform breeding strategies. Future research integrating multi-omics approaches with functional gene validation (importantly, in conditions where root growth and water uptake is not restricted by confounding factors such as pot binding) will be critical for dissecting these complex interactions, and for harnessing the potential of VOCs to develop more drought-tolerant potato varieties. This will help contribute to global food security in the face of increasing environmental challenges for potato production.

## Data Availability

The original contributions presented in the study are included in the article/supplementary material. Further inquiries can be directed to the corresponding author.
